# Barriers to and Facilitators of Older People’s Engagement With Web-Based Services: Qualitative Study of Adults Aged >75 Years

**DOI:** 10.2196/46522

**Published:** 2024-02-28

**Authors:** Annemarie Money, Alex Hall, Danielle Harris, Charlotte Eost-Telling, Jane McDermott, Chris Todd

**Affiliations:** 1 School of Health Sciences University of Manchester Manchester United Kingdom; 2 National Institute for Health and Care Research, Applied Research Collaboration Greater Manchester Division of Nursing Midwifery and Social Work, Faculty of Biology, Medicine and Health Manchester United Kingdom; 3 Manchester Institute for Collaborative Research on Ageing The University of Manchester Manchester United Kingdom; 4 Manchester University NHS Foundation Trust Manchester United Kingdom

**Keywords:** digital exclusion, digital inclusion, older people, technology, aged, web-based, internet

## Abstract

**Background:**

The COVID-19 pandemic has accelerated the shift toward the digital provision of many public services, including health and social care, public administration, and financial and leisure services. COVID-19 services including test appointments, results, vaccination appointments and more were primarily delivered through digital channels to the public. Many social, cultural, and economic activities (appointments, ticket bookings, tax and utility payments, shopping, etc) have transitioned to web-based platforms. To use web-based public services, individuals must be digitally included. This is influenced by 3 main factors: access (whether individuals have access to the internet), ability (having the requisite skills and confidence to participate over the web), and affordability (ability to pay for infrastructure [equipment] and data packages). Many older adults, especially those aged >75 years, are still digitally excluded.

**Objective:**

This study aims to explore the views of adults aged >75 years on accessing public services digitally.

**Methods:**

We conducted semistructured qualitative interviews with a variety of adults aged ≥75 years residing in Greater Manchester, United Kingdom. We also interviewed community support workers. Thematic analysis was used to identify the key themes from the data.

**Results:**

Overall, 24 older adults (mean age 81, SD 4.54 y; 14/24, 58% female; 23/24, 96% White British; and 18/24, 75% digitally engaged to some extent) and 2 support workers participated. A total of five themes were identified as key in understanding issues around motivation, engagement, and participation: (1) “initial motivation to participate digitally”—for example, maintaining social connections and gaining skills to be able to connect with family and friends; (2) “narrow use and restricted activity on the web”—undertaking limited tasks on the web and in a modified manner, for example, limited use of web-based public services and selected use of specific services, such as checking but never transferring funds during web-based banking; (3) “impact of digital participation on well-being”—choosing to go to the shops or general practitioner’s surgery to get out of the house and get some exercise; (4) “the last generation?”—respondents feeling that there were generational barriers to adapting to new technology and change; and (5) “making digital accessible”—understanding the support needed to keep those engaged on the web.

**Conclusions:**

As we transition toward greater digitalization of public services, it is crucial to incorporate the perspectives of older people. Failing to do so risks excluding them from accessing services they greatly rely on and need.

## Introduction

### Background

The shift toward digital technologies to provide access to essential and nonessential public services, such as health care, public administrative services, utilities, financial services, and leisure, has been accelerated by the COVID-19 pandemic [1-3]. In many countries, including the United Kingdom (the setting for the research reported in this paper), this shift has also been driven by national policy in attempts to maintain access to essential services during periods of social lockdown [4-6]. Many social, cultural, and economic activities, such as appointment or ticket booking, have transitioned to web-based platforms [7], whereas many COVID-19–related initiatives, such as booking and recording COVID-19 tests and vaccinations, were primarily offered through digital platforms to the public. In particular, during the COVID-19 lockdown periods, being on the web became critical for maintaining social ties and combating loneliness [8-10].

This may be convenient for many people, and there is evidence to show that digital inclusion, accelerated by the pandemic, has increased in recent years [11]. However, although in the United Kingdom, the “digital divide” (the gap between those who do and those who do not have access to new forms of information technology [12]) may have narrowed in recent years, the impact has not been felt equally and has widened for some groups [13,14]. The digital divide remains especially wide for older adults aged ≥75 years; this is further exacerbated by living alone, having a limiting long-term condition, and being financially susceptible [15,16]. An analysis of the English Longitudinal Study of Ageing data conducted early in the pandemic (June and July 2020) showed that 45% of adults aged 52 to 64 years and 41% of adults aged 65 to 74 years used the internet more since the COVID-19 outbreak, but only 24% of those aged >75 increased their use and 9% were using it less [17].

Digital exclusion, in its broadest definition [15], relates to three connected aspects: (1) access, that is, whether individuals have access to the internet at home or elsewhere; (2) ability, that is, having the skills and confidence to participate over the web; and (3) affordability, that is, the ability to pay for infrastructure (equipment) and adequate data packages [16]. In the United Kingdom, recent data from the national communications regulator (Ofcom) suggests that 6% of UK households do not have home internet access, but this figure rises to 26% of people aged ≥75 years [18]. It is also acknowledged that an additional 2 million households are experiencing financial difficulty, and this will likely increase given the cost-of-living crisis from 2021 to 2023 [19,20]. Digital exclusion is viewed as a “super” social determinant of health [21] as it impacts a variety of areas of life, including leading to poorer health outcomes [22,23] and challenges with employment, housing, education, and finance. It disproportionately affects many people, including people with low incomes, people living in social housing, people living with disabilities, people in rural areas, and people for whom English is not their first language, as well as other marginalized groups. Although all these factors are important indicators of who is likely to be digitally excluded, age remains the biggest indicator. According to data from the UK Office for National Statistics for 2020, a total of 99% of adults aged 16 to 44 years were recent internet users compared with only 54% of adults aged ≥75 years [24]. It is vital to understand the complexities of how digital exclusion exacerbates health and social inequalities so that adequate responsive action can be considered. For example, it is not simply the case that ensuring internet connectivity will mitigate digital exclusion. There is a need to understand structural challenges; financial barriers; digital literacy; and other aspects, such as the impacts of various health conditions and disabilities and concerns about privacy and data protection [25-28].

A recent scoping review explored the barriers to and facilitators of older people’s digital engagement across the spectrum of nonuse through sustained use [29]. This review found that there are substantial overlaps between barriers and facilitators; for example, lack of knowledge of digital technologies is a barrier, prior knowledge is a facilitator, perceived lack of personal capability is a barrier, and a positive attitude toward oneself is a facilitator. The review also found a substantial gap regarding the determinants of technological nonuse. Although this review provides a thorough scope of the literature, it included studies involving participants with a mean age of ≥65 years and did not offer any further stratification by age. There is a need for a more nuanced focus on older groups of older people. Current insights into digital technology use often exclude a specific focus on people aged ≥75 years. The coverage of age groups in several major reports and surveys often stops at the age of 74 years or includes all people aged ≥65 years as 1 homogeneous age category [30].

### Objectives

Given the increased risk of digital exclusion among older age groups and the fact that this may have been exacerbated by the COVID-19 pandemic, it is vital to gain a deeper understanding of the use, attitudes, and preferences of people aged ≥75 years.

In the United Kingdom, this gap has been recognized as a policy priority. In Greater Manchester, a city-region in North West England with a population of 2,867,800 in 2021, as many as 1.2 million residents are estimated to be limited digital users because of exclusion or personal preferences, with a substantial proportion of these being people in later life. To address this digital divide, the Greater Manchester Combined Authority (GMCA) established a Digital Inclusion Action Network and Taskforce in October 2020 [31], with the ambition to make Greater Manchester a 100% digitally enabled UK city-region. Older adults aged ≥75 years were included as a critical population group for targeted action related to digital inclusion. The aim of the paper is to report findings from a qualitative study exploring the views of adults aged ≥75 years on accessing web-based public services.

## Methods

### Study Design

The National Institute for Health and Care Research Applied Research Collaboration Greater Manchester was commissioned by the GMCA to gather insight into the barriers to and facilitators of older adults’ (>75 y) digital participation within the region. To address this, we conducted semistructured qualitative interviews with adults aged ≥75 years residing in Greater Manchester.

### Sampling, Recruitment, and Data Collection

Purposive and convenience sampling were used to identify and recruit participants. Adapting to web-based rather than face-to-face data collection during the COVID-19 pandemic lockdown meant that we had to use an approach that allowed us to use our existing networks and recruit via several third-party organizations affiliated with the GMCA Ageing Hub. We recruited a range of older adults; these included older adults who were fully engaged and participating in many web-based activities (often enrolled in a local support program), those who were just starting to receive support to get on the web, those who were previously engaged but were now lapsed users, and those who had no interest in getting on the web or using a computer or device at all. We also recruited community support workers to learn from the approaches they used to continue to engage with their communities, particularly during the COVID-19 pandemic period (from 2020 to 2022) [32].

Data were collected via semistructured interviews (conducted by AM) with a topic guide (Multimedia Appendix 1) developed from a rapid review of the literature [33] and from input from the project oversight team.

### Data Analysis

Interviews were audio recorded, transcribed, and exported to NVivo Pro (version 12) software for data management [34]. Using a thematic analysis approach [35], initial themes were identified from the transcripts and indexed to develop the analytical categories. Via a process of constant comparison [36], these categories were reviewed and refined by 2 researchers (AM and DH), and any ambiguities in the coding framework were reconciled by a thorough discussion with the research team. All interviews were then fully coded using NVivo Pro for qualitative analysis (AM and DH).

### Ethical Considerations

Ethics approval was granted by the University of Manchester Proportionate Research Ethics Committee (2021-12638-20811). All interviews were conducted virtually (by telephone or other remote means agreed upon with the participants) at a time convenient to the participants; the participants provided informed consent before data collection. The data were collected between October 2021 and February 2022. All data provided was anonymised by the research team with any personal identifying information removed. All participants received a £15 'Love2Shop' voucher as a thank you for their participation in the interview.

## Results

### Overview

The final sample comprised 26 interviews: 92% (24/26) with older adults aged >75 years and 8% (2/26) with community digital support officers. The older adult sample had a mean age of 81 (SD 4.54; range 75-91) years; 58% (14/24) of the participants were female, 96% (23/24) were White, and 4% (1/24) were of South Asian background. Participants were sampled from 4 (40%) of the 10 local authority areas in Greater Manchester, 75% (18/24) of the participants were users of the internet (to some degree), and interviews lasted on average 23 (SD 8.51; range 8-60) minutes.

A total of 5 themes were identified as being key in understanding the barriers to and facilitators of motivation, engagement, and participation in using web-based public services. The key themes and subthemes are presented in Textbox 1 and discussed in detail in the subsequent sections.

**Key themes and subthemes identified via thematic analysis.**

- **Theme 1: initial motivation to participate digitally**
- **Theme 2: narrow use and restricted activity on the web**
  - Preference for choice of access
  - Narrow use driven by fear
  - Lack of interest in learning new digital skills and tasks
- **Theme 3: impact of digital participation on well-being**
- **Theme 4: the “last generation”?**
- **Theme 5: making digital accessible**
  - One-to-one support
  - Mitigating physical impairments

### Theme 1: Initial Motivation to Participate Digitally

Among older adults who were using the internet, it was evident that their decision to do so was often driven by a particular, recent need that motivated them to go on the web. Unsurprisingly, given that COVID-19 pandemic lockdowns reduced face-to-face social contact [32], one of the major motivators related to maintaining social connections and gaining skills to be able to connect with family and friends in other parts of the world. A participant stated the following:

Well, I think that’s vital really [being online]. It’s kept me alive in that, you know, I feel as if I’m speaking to people. It’s company there. I’m never isolated because I can always get in touch with somebody. So, to me, it has literally been a lifeline.

Participant 19, female, aged 82 years

There was also the need and convenience of being able to access certain services during the lockdown, in particular web-based ordering of prescriptions and shopping. A participant said the following:

That’s the most brilliant thing I’ve ever used, Amazon. You don’t even have to go outside the door.

Participant 13, female, aged 75 years

### Theme 2: Narrow Use and Restricted Activity on the Web

#### Preference for Choice of Access

Although three-quarters (18/24, 75%) of the older adults interviewed were digitally engaged (to some extent), many of them were “narrow” users [11,15], in that they participated only in a handful of web-based activities or tasks. The participants reported very little interaction with web-based public administrative services (eg, local and national government services such as disabled parking applications, passport applications, driving licenses, and benefit applications). The participants were more positive about some aspects of web-based health services, particularly ordering prescriptions. When asked why they preferred to order prescriptions over the web, some commented that the system is “straightforward to use” and that they “find it very useful” to be able to order over the web, with some noting that if they did not do it over the web, it would involve them going to the surgery, “which is a bus ride away.” However, when asked about the prospect of more public services moving to web-based access, most participants—both those who used the internet and those who did not—were in consensus that digital should not be the only option provided by organizations to access a service, for a variety of reasons. A participant stated the following:

The jabs that I had, it was telling me to go online, that’s an example, and I phoned up my doctor’s surgery and said, look, I can’t go online, right, so they did the appointment for me. And also I had a bit of an argument with [large retail pharmacist named] and other stores like [pharmacy chain] because the flow thing, you know the flow thing, [Lateral Flow Test- rapid antigen test for COVID-19] you’ve got to go online although you didn’t get any because they were sold out, they were out of them all the time...it’s just ridiculous, nobody thinks about the older people.

Participant 14, male, aged 83 years

Another participant said the following:

Personally I don’t think you can beat seeing the doctor face to face, they can pick up on your body language, colour of your skin. I think there’s lots of things that you can pick up on face to face. So, I do think seeing the doctor face to face is essential for the majority, I really do. I think going online for some things is good, but I do think if you’re not very sure about what you’re doing, I think...I would imagine it could cause a lot of stress, if there’s no alternative...

Participant 24, female, aged 76 years

#### Narrow Use Driven by Fear

Many participants adopted a granular approach to use, in which they had specific and limited web-based tasks they would undertake within particular domains of activity, such as banking or shopping. For example, many were happy to log on to banking apps or websites and view their balance—that is, to monitor their account—but stopped short of undertaking any transactions. Reasons for this limited use included a concern about having personal details “out there,” pressing the wrong button and sending the money to the wrong place, and a fear of being scammed. A participant stated the following:

...No, I won’t do finance at all, PayPal or anything, I really don’t trust it because there are so many scams around, erm, I just think it’s too easy, if you press the wrong button and its gone to Timbuktu, no I definitely won’t have anything to do with online banking, and it’s a shame because I know that I would shop online and it would save a trip to the Post Office or the bank or whatever, but I just wouldn’t trust it...

Participant 7, female, aged 79 years

Another participant stated the following:

...I know I’ve got the banking online on the tablet if I want to use it for transactions, but I’m just quite happy seeing what I’ve got at the moment. I don’t really feel confident enough to do transactions. I always worry, God if I do something wrong, I’m in trouble.

Participant 24, female, aged 76 years

In addition, another participant said the following:

I just feel as though I don’t want to be divulging too much information about myself to the wide world, if you know what I mean.

Participant 11, female, aged 87 years

Community digital support officers highlighted fear and concerns around the safe sharing of personal information as a key barrier to engagement among older adults. In particular, media reports highlighting scams and frauds were deemed to exacerbate this barrier. The community officers were aware of the need to inform people of the potential risks, but “more positive campaigns about [the benefits of] using the internet [for older people] are needed” (digital support officer 2), as the negative stories reported on television were seen to deter older adults from benefiting from available web-based services. They reported that the word “scam” really “puts fear into older persons” and the media “cherry pick” the very worst scams to the point that people are convinced that these are happening on their very doorsteps:

...And this is no joke, I have had people say to me that they think there are people outside their house on a laptop in their car, you know they are parked on the street trying to use their Wi fi to scam them. That sort of thing.

Digital support officer 1

#### Lack of Interest in Learning New Digital Skills and Tasks

Where participants did use web-based services, once their initial needs had been met and they had gained the necessary skills to complete an activity, many lacked interest or were reluctant to undertake additional tasks or learn new activities. They were happy to maintain the skills and knowledge gained to undertake the tasks or activities that initially prompted them to get on the web. A participant stated the following:

I wouldn’t use it for much really. I’m not ambitious about it. I have done what I wanted to do and anything else that I gained, it’s a bonus.

Participant 10, female, aged 91 years

Another participant stated the following:

Are there any tasks that you haven’t yet done online that you think you might want to try or you want to do in the future?

Interviewer

No, because I can use the computer and I can use the phone and the tablet for anything that I personally need to do.

Participant 20, male, aged 76 years

In addition, another participant said the following:

And are there things that you would want to do, that maybe you don’t yet know how to do, or you’d need some support to be shown how to do it?

Interviewer

I don’t think so. I think I do what I need to do...

Participant 13, female, aged 75 years

### Theme 3: Impact of Digital Participation on Well-Being

For some participants, who were not digitally engaged and had no interest in getting on the web, social connections and social interaction were cited as a reason for not engaging. One participant stated the following:

No, I just think I’ve never been interested. I feel that if I did use something I’d be on my own doing it, and I don’t like being on my own. Years ago I had one of these knitting machines and I had it for a while, and I hated it because it meant I was sat on my own knitting, and I don’t want to do that. I like to go out and meet people while I can.

Participant 3, female, aged 90 years

Another participant stated the following:

This is the problem, lots of people don’t talk anymore. They know...they don’t know any other way of corresponding, getting in touch with people. I mean they go on the internet. They text, they don’t talk...I mean you go out for a walk and you can more or less guarantee at least 50 per cent of people walk around with their phone.

Participant 5, male, aged 83 years

The participants also spoke about digital engagement in relation to aspects of physical and mental well-being. For example, for some, not using web-based services, such as to make a general practitioner (family physician in the United Kingdom) appointment, was seen as a positive because they had to get “out of the house” and, in doing so, had the benefit of getting “a little bit of exercise.” For others, there was the acknowledgment of the advantages of being able to shop over the web during lockdowns, but now that restrictions had lifted, they had reverted to their preference to shop in person, which again was seen as an opportunity for exercise. Others talked about how it was sometimes “too easy” to depend on the internet to find out information that they could not immediately call to mind, and this was spoken about in terms of brain health and keeping the mind active. A participant stated the following:

So, in terms of doctors’ appointments and things, you can still get to the surgery or you could ring. Is that something that you prefer to do?

Interviewer

Yes, I can ring on my landline and talk to a receptionist, or just toddle myself down to the surgery and go face to face with them, you know...Not that I would get an appointment any quicker with the doctor but, you know...And it gives me a little bit of exercise.

Participant 18, female, aged 79 years

Another participant stated:

I’ve gone back to going out because you get a little bit of exercise, you know. So yeah, I don’t shop for groceries online anymore, no, I always go to the shop.

Participant 20, male, aged 76 years

Another participant stated the following:

...A couple of days ago, it sounds ridiculous this...I thought, what’s the name of that pub at the top of Lancashire Road? I mean, what the hell I thought about that for, and I could not for the life of me, and I thought, no...remember it, because you do know it. And this morning, it’s come to me, The Hinds Head it’s called.

Participant 13, female, aged 75 years

Okay, so you resisted the urge to find out?

Interviewer

I did, yes, I did. I thought, no, that’s too easy.

Participant 13, female, aged 75 years

### Theme 4: The “Last Generation”?

Many participants, particularly those who were not on the web, spoke about barriers regarding generational issues and how they felt they might be the “last generation” to experience difficulty with digital participation:

But I do think that we’re the last generation, almost the last generation that this will affect. Because from being babies now they have iPads now and what-have-you, don’t they? It’s just second nature to them. It puts you to shame when you watch them.

Participant 12, female, aged 81 years

This often went hand-in-hand with the perception that these difficulties were unique to their generation and that the younger generations experienced little difficulty in adapting to or embracing new technology. A participant stated the following:

Well, do you know what, to be honest with you, I could say I’m at the end of a generation. Because if you think of the youngsters today now and you think of...like my sons have no problem with this, that’s another generation, and then the one below that is the youngsters, yeah, this will never happen again...it’s unlucky, I’m at the end of a generation.

Participant 14, male, aged 83 years

Another participant stated the following:

The youngsters, from school onward, they know nothing but the internet. So everything is being geared toward them. And we older people, in my generation, have had to start learning various things which become harder and harder. It’s second nature to younger people, to the 30s, 40s.

Participant 5, male, aged 83 years

### Theme 5: Making Digital Accessible

For those older adults who were participating (to some extent) in web-based activities, it was important to discuss barriers and facilitators that might require consideration to keep older adults engaged and supported.

#### One-to-One Support

Participants emphasized the importance of having patient assistance while navigating tasks on the web. They also highlighted the value of receiving written instructions and having tasks demonstrated multiple times. The participants also valued the one-to-one support given to them but stressed that this needed to be ongoing support, noting that sometimes they would “get the hang of” one task (eg, shopping) only to find that the next time they logged on to the website, the landing page may have changed, which would “throw them off” and result in them feeling unsure whether they could continue in the manner they had been shown. A participant stated the following:

I’d love someone to sit and show me so I can write it down and if I get stuck I know how to do it myself.

Participant 19, female, aged 82 years

Other participants stated the following:

She writes things down for me, because I can’t always remember what I’ve been told. If I’ve got it there in black and white then I can follow it. It does help.

Participant 2, female, aged 76 years

Yeah, sometimes it doesn’t click immediately and you need them to go over it again. So you need somebody who’s got a little bit of patience.

Participant 21, male, aged 78 years

...I could do this before and now I’m having too many problems. And it’s the same with...so what it is, is what they call navigating the website becomes more difficult when they change the format, and that I find very, very annoying.

Participant 8, male, aged 75 years

You know, the system I have for my laptop, when they start changing things I get very annoyed and I think, oh, I’ve got to figure out how to get out of that or whatever it is, yes...I’m just getting really annoyed when I have to figure out how all of these things work again.

Participant 1, female, aged 76 years

#### Mitigating Physical Impairments

In addition, we asked respondents about physical impairments that might currently (or potentially in the future) make digital participation difficult. Arthritis, cataracts, Parkinson disease, diabetes, and tremors were all listed as having an effect on current internet use. Regarding future impact, although acknowledging uncertainty about how this could develop—“my eyes are not great. Yeah, I don’t know how that’s going to go*.*” (participant 1, female, aged 76 y)—many of the respondents were quick to point out potential solutions to overcome these; for example, some had already been shown how to locate and use the microphone function in Google Assistant, how to use predictive text, and how to increase the font size of the text on the screen. Many respondents had already taken these issues into consideration when deciding on the type of device to use. A total of 61% (11/18) preferred to use tablets, and this was for several reasons, including their portability and ease of use. One participant stated the following:

I also like the fact that I can have it on my knee in the lounge or the chair that I’m in.

Participant 19, female, aged 82 years

## Discussion

### Principal Findings

The aim of this project was to explore the views of adults aged ≥75 years on accessing web-based public services in response to a policy initiative to further understand older people’s digital behavior and engagement. The analysis of the semistructured interviews identified 5 themes that were key to understanding some of the barriers and facilitators experienced by the older adults participating in this project. The key facilitators included responses to meeting certain needs (particularly during national lockdowns) such as food shopping, ordering prescriptions, and staying connected with family and loved ones. The identified barriers included fear of scams and misuse of personal information, lack of ongoing support to maintain or learn new skills, preference for face-to-face interactions (especially for health appointments), and a wider generational belief (held by many) that difficulties getting on the web were “unique” to their generation and that older adults found it difficult to adapt or embrace new technology. Crucially, we also found that the potential unintended consequences of the benefits offered by digital technologies to access public services could be seen as a barrier to their use. In particular, this included their ease of access to information and their convenience, which were seen to reduce the need for people to engage cognitively elsewhere or to leave the house, thereby denying them exercise and social interaction opportunities.

Theoretically, there are several models that attempt to explain digital engagement and uptake. Two of the most well-known and widely used are the Technology Acceptance Model [37] and the Unified Theory of Acceptance and Use of Technology [38]. However, these models are primarily used to quantify the acceptance of technologies rather than to provide qualitative insights [29]. One straightforward categorization to facilitate an understanding of different “types” of older adults’ digital behavior suggests they may fall into 1 of 4 groups—“engaged,” “disheartened,” “transitional,” or “uninterested” [39]. “Engaged” refers to those older adults who believe they are capable of learning and perceive a value in using digital technology, that is, believe the internet is useful to them personally. “Disheartened” users also believe the internet to be useful and usually have more need for digital services but are worried about safety and associated risks and feel less confident in their ability and skills. “Transitional” older adults often have the highest need for use of digital services, but they are frequently lapsed users with narrow, if any, experience of digital engagement other than for social media purposes. “Uninterested” older adults do not perceive value in web-based activity and often have others access websites on their behalf. They usually have strong social connections and can be resistant to using the web. Although these categories are helpful in starting to think about digital behavior and potentially offer insights into how to support different “types” of older adults, the interviews presented here show that older adults’ digital behavior may not always be neatly classified into 1 type of user versus another. A large proportion of the respondents could be classified as digitally “engaged” in the sense that they were keen to go on the web, felt capable of learning, and had many of the skills deemed “essential” [40] for digital participation.

However, it was not possible to map these older adults to 1 “type” as there was often an overlapping of categories (particularly “engaged” and “disheartened”), which required a more nuanced understanding of what digital participation means for older adults. During discussions around the motivations behind getting on the web for our older adults (particularly during the pandemic), the initial “engagement” was evident; however, this engagement for many appeared to ebb away, and subsequently, many of them fitted the descriptions for other categories, for example, “disheartened” users. Although many of our older adults admitted a perceived value in accessing the internet and participating digitally (ie, “engaged”), a lack of confidence, lack of support, or fear of sharing information would often result in them becoming “disheartened,” disengaging from aspects of internet use, and not taking full advantage of the services available to them (eg, restricted use of web-based banking). Understanding that many older adults will not “fit” into 1 category highlights the need for a more individualized and nuanced approach to tailoring digital support services [41].

When considering the wider impacts of digital technology transformation, older adults’ limited use of web-based activities such as banking and concerns over data protection were also amplified by a lack of confidence and skills. Added to this were the needs of older people to get out and to socialize, with health-related appointments, shopping, and banking forming part of how participants stayed active and well in their communities. Work undertaken with a range of individuals, including older adults, during the pandemic found that a move to internet-based general practitioner and health appointments was sometimes problematic for this group for a variety of reasons, including a lack of skills and confidence, no interest in engaging on the web, and a lack of trust [42-44]. In addition, web-based platforms provide a very limited 2D view of a person and their circumstances [45,46]. As such, it is critical that people are encouraged to leave their homes and are able to access and attend face-to-face appointments. We already know that far too many older people are sedentary and do not achieve the recommended levels of physical activity [47,48]. This worsened during the COVID-19 pandemic, negatively impacting health [49]. Therefore, adding to this burden by substituting physical activity with digital engagement should be avoided.

The pandemic and its successive lockdowns have moved much public and social activity to web-based platforms. Digital exclusion is often discussed in terms of “hard” (eg, never having used the internet or having no internet access) or “soft” (eg, improving digital engagement, skill level, or confidence) [7]. These changes driven by the pandemic are said to have improved “hard” exclusion for the general population. However, in terms of improving the “softer” areas of exclusion, the pandemic has done little to close the digital divide, particularly for older adults [50]. There is evidence to show rates of internet use increasing faster among younger cohorts and declining among older cohorts, demonstrating the digital divide naturally closing in time as generations who experience high levels of digital exclusion are replaced by younger generations who embrace and adapt to technological change [51,52]. This idea came through strongly in the interviews conducted, with many older adults expressing this view. However, it was not clear whether they attributed this to the impact of the rapid digitalization brought about by the pandemic or to the impact of a more general move toward digitalization over a longer period. However, the rapid development of technology combined with an individual’s changes in physical health has been shown to worsen feelings of being unable to keep up or feeling too old to embrace new technology among older adults [53]. There is also the view that older adults can often internalize agism [54] and accede to the stereotype that they are not able to master technology and so do not attempt it. It seems plausible that the rapid increase in digitalization during the pandemic may have exacerbated these feelings of being left behind, but it is also important to note that inaccurate perceptions of young people as fluent technology users may be driven by a broad range of factors, including media representation, agism, and other social constructs related to digital inclusion and exclusion. Although the impact of the 2020 to 2023 COVID-19 pandemic brought many of these issues to the forefront in discussions around digital participation and the impact of the divide for older adults, these are not new issues related only to older adults’ experiences during the pandemic [50].

This study highlights several digital technology features that have delivered positive outcomes for people aged ≥75 years. Among those we interviewed, there was a preference toward using tablets [55] as well as a willingness to order prescriptions and engage in web-based shopping. Shared learning across public institutions on aspects of digital technology transformation that have been delivered successfully for adults aged >75 years would be beneficial. For example, what can we learn from the experience of web-based ordering of prescriptions that could inform other public service digitalization infrastructure and processes? There is also the importance of ensuring digitalized services are fully accessible to all, including those living with long-term health conditions that may impact their ability to use digital devices (eg, arthritis) or access content easily (websites, text, etc). For older adults who wish to participate on the web, building confidence in undertaking tasks, such as banking, via support that is task focused and repeated is crucial [56,57].

### Strengths and Weaknesses

Current data and insight into digital technology use may exclude people aged ≥75 years or may lack a specific focus on this age group. Often, available data on older adults’ use stops around the age of 74 years or it provides information on all individuals aged ≥65 years. Given the increased risk of digital exclusion among older age groups and the fact that this may have been exacerbated by the COVID-19 pandemic, it is vital to gain a deeper understanding of the use, attitudes, and preferences of people aged >75 years. Although this research was relatively small in number, its strength is that it focuses on those aged >75 years (average respondent aged 81 years). A key message to take away from the work is one of choice: that those aged ≥75 are not digitally homogenous but rather require a range of options, both digital and nondigital, that will enable them to engage in ways that work best for them and do not further exacerbate digital inequalities [23,58-60]. A key finding adding to the literature [29] is that the benefits of technologies, such as ease of access to information and convenience, may actually have unintended consequences that put older adults off using them. This includes a desire among some participants to continue to access some public services in person for the indirect benefit of physical activity while doing so. This finding is particularly important in light of other healthy aging policies that promote physical activity to improve disability-free life expectancy [61].

Future research should investigate the experiences of older adults from ethnic minority groups. Although this study aimed to be as inclusive as possible, the recruitment of older adults from diverse ethnic minority groups proved challenging. It would also be beneficial to examine the influence of age-related sensory changes on digital inclusion. Investigating the preferences and experiences of older adults with hearing or visual impairments would provide valuable insights. Physical distancing and stay-at-home restrictions during pandemic lockdowns meant that our recruitment strategy had to be adapted to make use of our existing networks and ties with third-party organizations to be able to recruit older adults for the study. A reliance on web-based means of recruitment resulted in a more digitally engaged sample of older adults being recruited than originally intended, although the levels of engagement varied among the older adults. In an ideal setting, a study of this nature would have been conducted with older adults in a face-to-face setting rather than via telephone or video interviews [62-64]. However, data collection during the COVID-19 pandemic meant this approach was not possible, and this will have had an impact on the final sample of older adults, with a larger proportion of adults who were digitally engaged taking part.

### Conclusions

The shift to digital delivery of public services, both throughout the pandemic and more generally as a driving force for future service provision, requires a focus on the needs and preferences of older people so that they are not excluded from service access. Mitigation against digital exclusion is a core component of 1 of the strategic priorities to reduce health inequalities across England [52]. It is vital that the needs and preferences of people of all ages are considered, particularly those aged >75 years, who are often underrepresented in research. Attempts to classify “types” of digital users may be a useful heuristic for thinking about digital engagement, but the boundaries between categories are permeable and complex. Those aged ≥75 years are not a digitally homogenous group but rather require a range of options, both digital and nondigital, that will enable them to access services without further exacerbating digital inequalities.

## Appendix

Multimedia Appendix 1 Interview topic guides.

## Abbreviations

GMCA: Greater Manchester Combined Authority

## References

[1] (2021). Global strategy on digital health 2020-2025. World Health Organization.

[2] Contreras RR Covid-19 and digitalisation. Eurofound.

[3] (2020). OECD Digital Economy Outlook 2020. Organisation for Economic Cooperation and Development.

[4] (2019). NHS long term plan. National Health Service.

[5] (2021). Build Back Better: our plan for growth (HTML). United Kingdom Government.

[6] (2014). Transforming public services, using technology and digital tools and approaches. Local Government Association.

[7] Abey J (2022). Bridging the divide: tackling digital inequality in a post-pandemic world. The Fabian Society.

[8] Boulton E, Kneale D, Stansfield C, Heron P, Sutcliffe K, Hayanga B, Hall A, Bower P, Casey D, Craig D, Gilbody S, Hanratty B, McMillan D, Thomas J, Todd C (2021). Rapid systematic review of systematic reviews: what befriending, social support and low intensity psychosocial interventions, delivered remotely, may reduce social isolation and loneliness among older adults and how?. F1000Res.

[9] (2021). COVID-19 and the digital divide. Centre for Ageing Better.

[10] Savage RD, Di Nicolo S, Wu W, Li J, Lawson A, Grieve J, Goel V, Rochon PA (2022). The factors associated with nonuse of social media or video communications to connect with friends and family during the COVID-19 pandemic in older adults: web-based survey study. JMIR Aging.

[11] Lythreatis S, Singh SK, El-Kassar AN (2022). The digital divide: a review and future research agenda. Technol Forecast Soc Change.

[12] van Dijk JA (2006). Digital divide research, achievements and shortcomings. Poetics.

[13] (2021). Digital inclusion and older people – how have things changed in a Covid-19 world?. Age UK.

[14] Building a digital nation. Good Things Foundation.

[15] Carney F, Kandt J (2022). Health, out-of-home activities and digital inclusion in later life: implications for emerging mobility services. J Transport Health.

[16] Gallistl V, Rohner R, Hengl L, Kolland F (2021). Doing digital exclusion - technology practices of older internet non-users. J Aging Stud.

[17] Kung CS, Steptoe A (2023). Changes in internet use patterns among older adults in England from before to after the outbreak of the COVID-19 pandemic. Sci Rep.

[18] (2022). Digital exclusion: a review of Ofcom’s research on digital exclusion among adults in the UK. Ofcom.

[19] UK digital poverty evidence review 2022. Digital Poverty Alliance.

[20] Hourston P (2022). Cost of living crisis. Institute for Government.

[21] Sieck CJ, Sheon A, Ancker JS, Castek J, Callahan B, Siefer A (2021). Digital inclusion as a social determinant of health. NPJ Digit Med.

[22] Lu X, Yao Y, Jin Y (2022). Digital exclusion and functional dependence in older people: findings from five longitudinal cohort studies. EClinicalMedicine.

[23] Seifert A, Cotten SR, Xie B (2021). A double burden of exclusion? digital and social exclusion of older adults in times of COVID-19. J Gerontol B Psychol Sci Soc Sci.

[24] (2021). Internet users, UK: 2020. Office for National Statistics.

[25] Davies AR, Honeyman M, Gann B (2021). Addressing the digital inverse care law in the time of COVID-19: potential for digital technology to exacerbate or mitigate health inequalities. J Med Internet Res.

[26] Yang E, Lee KH (2022). The moderating effects of disability on mobile internet use among older adults: population-based cross-sectional study. J Med Internet Res.

[27] Poulsen A, Song YJ, Fosch-Villaronga E, LaMonica HM, Iannelli O, Alam M, Hickie IB (2023). Digital rights and mobile health in low- and middle-income countries: protocol for a scoping review. JMIR Res Protoc.

[28] Heponiemi T, Jormanainen V, Leemann L, Manderbacka K, Aalto AM, Hyppönen H (2020). Digital divide in perceived benefits of online health care and social welfare services: national cross-sectional survey study. J Med Internet Res.

[29] Kebede AS, Ozolins LL, Holst H, Galvin K (2022). Digital engagement of older adults: scoping review. J Med Internet Res.

[30] ICT usage in households and by individuals (isoc_i). Eurostat.

[31] New Digital Inclusion Taskforce launched to tackle digital divide across Greater Manchester. Greater Manchester Combined Authority.

[32] Sherrington A (2022). 2 years of COVID-19 on GOV.UK. United Kingdom Government.

[33] Hall A, Money A, Eost-Telling C, McDermott J (2022). Older people's access to digitalised services - a rapid literature review. National Institute for Health and Care Research.

[34] NVivo. Lumivero.

[35] Boyatzis RE (1998). Transforming Qualitative Information: Thematic Analysis and Code Development.

[36] Pope C, Ziebland S, Mays N (2000). Qualitative research in health care. Analysing qualitative data. BMJ.

[37] Davis FD, Davis F (1989). Perceived usefulness, perceived ease of use, and user acceptance of information technology. MIS Q.

[38] Venkatesh V, Morris MG, Davis GB, Davis FD (2003). User acceptance of information technology: toward a unified view. MIS Q.

[39] I am connected: new approaches to supporting people in later life online. Good Things Foundation.

[40] (2021). Essential digital skills UK 2021. Ipsos.

[41] Schroeder T, Dodds L, Georgiou A, Gewald H, Siette J (2023). Older adults and new technology: mapping review of the factors associated with older adults' intention to adopt digital technologies. JMIR Aging.

[42] Tewary S, Cook N, Dezine M, Shnayder O, Pandya N (2023). Supporting vulnerable older adults with telehealth through wellness calls and tablet distribution during COVID-19: quality improvement project. JMIR Form Res.

[43] Wilson J, Heinsch M, Betts D, Booth D, Kay-Lambkin F (2021). Barriers and facilitators to the use of e-health by older adults: a scoping review. BMC Public Health.

[44] Frishammar J, Essén A, Bergström F, Ekman T (2023). Digital health platforms for the elderly? key adoption and usage barriers and ways to address them. Technol Forecast Soc Change.

[45] Parsons L (2021). Study reveals lack of diagnostic accuracy in online consultations. PharmaTimes.

[46] Kendrick K (2020). As telemedicine replaces the physical exam, what are doctors missing?. National Public Radio.

[47] (2019). Physical activity guidelines: UK Chief Medical Officers' report. Department of Health and Social Care United Kingdom Government.

[48] Skelton DA, Mavroeidi A (2018). How do muscle and bone strengthening and balance activities (MBSBA) vary across the life course, and are there particular ages where MBSBA are most important?. JFSF.

[49] Elliott J, Munford L, Ahmed S, Littlewood A, Todd C (2022). The impact of COVID-19 lockdowns on physical activity amongst older adults: evidence from longitudinal data in the UK. BMC Public Health.

[50] Zapletal A, Wells T, Russell E, Skinner MW (2023). On the triple exclusion of older adults during COVID-19: technology, digital literacy and social isolation. Soc Sci Humanit Open.

[51] Matthews K, Nazroo J, Marshall A (2018). Digital inclusion in later life: cohort changes in internet use over a ten-year period in England. Ageing Soc.

[52] (2021). Global report on ageism. World Health Organization.

[53] Pirhonen J, Lolich L, Tuominen K, Jolanki O, Timonen V (2020). “These devices have not been made for older people's needs” – older adults' perceptions of digital technologies in Finland and Ireland. Technol Soc.

[54] (2022). Ageism in artificial intelligence for health. World Health Organisation.

[55] Kim S, Yao W, Du X (2022). Exploring older adults' adoption and use of a tablet computer during COVID-19: longitudinal qualitative study. JMIR Aging.

[56] Doing digital in later life: a practical guide. Good Things Foundation.

[57] Hunsaker A, Nguyen MH, Fuchs J, Djukaric T, Hugentobler L, Hargittai E (2019). “He explained it to me and I also did it myself”: how older adults get support with their technology uses. Socius.

[58] Mubarak F, Suomi R (2022). Elderly forgotten? digital exclusion in the information age and the rising grey digital divide. Inquiry.

[59] Kunonga TP, Spiers GF, Beyer FR, Hanratty B, Boulton E, Hall A, Bower P, Todd C, Craig D (2021). Effects of digital technologies on older people's access to health and social care: umbrella review. J Med Internet Res.

[60] Quan-Haase A, Williams C, Kicevski M, Elueze I, Wellman B (2018). Dividing the grey divide: deconstructing myths about older adults’ online activities, skills, and attitudes. Am Behav Sci.

[61] Bull FC, Al-Ansari SS, Biddle S, Borodulin K, Buman MP, Cardon G, Carty C, Chaput JP, Chastin S, Chou R, Dempsey PC, DiPietro L, Ekelund U, Firth J, Friedenreich CM, Garcia L, Gichu M, Jago R, Katzmarzyk PT, Lambert E, Leitzmann M, Milton K, Ortega FB, Ranasinghe C, Stamatakis E, Tiedemann A, Troiano RP, van der Ploeg HP, Wari V, Willumsen JF (2020). World Health Organization 2020 guidelines on physical activity and sedentary behaviour. Br J Sports Med.

[62] Saarijärvi M, Bratt EL (2021). When face-to-face interviews are not possible: tips and tricks for video, telephone, online chat, and email interviews in qualitative research. Eur J Cardiovasc Nurs.

[63] Keen S, Lomeli-Rodriguez M, Joffe H (2022). From challenge to opportunity: virtual qualitative research during COVID-19 and beyond. Int J Qual Methods.

[64] Davies L, LeClair KL, Bagley P, Blunt H, Hinton L, Ryan S, Ziebland S (2020). Face-to-face compared with online collected accounts of health and illness experiences: a scoping review. Qual Health Res.

