# Supplementary material for: Barriers to and Facilitators of Older People’s Engagement With Web-Based Services: Qualitative Study of Adults Aged >75 Years
Source: JMIR Aging. 2024 Feb 28;7:e46522. doi: 10.2196/46522 (PMC10938227; doi:10.2196/46522)
Supplement: Multimedia Appendix 1 [file aging_v7i1e46522_app1.pdf]

What factors have impacted on older peoples (75+) access to and engagement with digital public services?

Draft interview topic guides

NB - 3 different interview guides for the 3 participant groups: users, non-users of digital services and community leaders (some questions may overlap)

1. Adopters / users –

Interview will start by explaining what we mean by digital public services, e.g. those Government based services that you apply for, renew or access online – examples include applying for benefits, renewing passports or driving licences, completing tax returns, paying council tax etc.

Ice breaker questions – recruitment for those who have got online will be via a local digital programme, so the first few questions would explore how they got involved in this, how long have they used it, were they encouraged by family / friend etc.

Explore past use of computers / smartphones / any other digital device, e.g. in work setting – if previously used digital services but then stopped or lapsed, why do they think that happened?

Exploration of what exactly they use the internet for (e.g. is it information gathering, do they email family, do they bank online, online shopping e.g. buy food, pay bills, access health)

Exploration of what they wouldn't use the internet for and why that is the case - so depending on answer above, probe for why some services over others? (Worries over security, remembering passwords, experience of scams, link in with older people being narrow users of the internet)

Any additional tasks they now undertake that they didn't initially think they would; prompts for why? Encouragement from family, friends, peers?

Ideas about what is beneficial about being digitally engaged, what is the internet good for? (Information at your fingertips, convenience, saves times, saves queuing in shops, saves petrol travelling to shops etc.)

Explore issues around convenience / relevance and value of internet use to their everyday lives?

Explore perceptions of older age and internet use – positive .v. negative ideas. Once over initial hurdle/ barrier, then becomes lot easier/ familiar.

Are physiological problems an issue? E.g. poor eyesight, tremors, hearing difficult etc…

Importance of continuing support and encouragement, and where that comes from (e.g. family / peers / tech programmes), which is best?

2. Non-adopters –need to start these interviews by teasing out whether participant has any interest in getting online but factors are hindering this, or whether they are totally resistant to getting online – this is important for some older people and we need to be careful to make sure that we acknowledge that its fine if that is the case, we just are interested in exploring why?

Explore past use of computers / smartphones / other digital device, e.g. ever used in a work setting? Prompt for reasons as to why this might have lapsed / why no longer, what has changed?

Explore reasons why they don't use it now – prompt for practical reasons (physical health reasons, cost / skills / equipment) prompt for security reasons (e.g. scams, scared of getting details stolen) prompt for other reasons (traditional approach to life, just not interested etc)

If you needed something that could only be purchased or applied for online, for example, a blue badge, how would you go about this? Do you have friends or family that access these services on your behalf? If not, then how would you get support to help you with these tasks?

Are they aware of places to go to if they did want to get online and needed support to do this?

Explore issues around convenience / relevance and value of internet use to their everyday lives? For example, potential time and money savings from internet banking, shopping, paying bills online, etc.

Explore perceptions of older age and internet use – positive .v. negative ideas

Explore resistance to change / technophobia?

Do they feel they are missing out by not being online?

Are physiological problems an issue? E.g. poor eyesight, tremors, hearing difficult etc…

Is there anything that might encourage use? Encouragement and help from family members? Probe for buddying / cheap or free equipment / being able to contact someone for real time help / other factors that might encourage use / or nothing at all that will encourage use?

Any views on the idea that you are in some way losing out if you are not using the internet?

3. Community leaders –

Explore background and current role; how long in role, what their current job requires etc

Explore specific role in relation to older adults, how has this worked during lockdown?

Has it been easy to obtain funding to purchase, lease computers (I.T equipment) for your group of older people? How was this achieved?

How have you tried to engage/support older members of your community in getting online? What if any resistance do you encounter from people, and why? Is language an initial barrier?

What are the main kinds of support that people seem to need in order to get online/use online services? Why do you think this might be the case? To what extent do you think that support needs have changed since the COVID pandemic?

What are the common barriers to getting online among members of your community? Are there any noticeable differences among the males/ females?

Have you managed to encourage anyone who has been reluctant to get online? If so, how have you done this/what approaches do you find most successful?

Would you say that once the initial barrier(s) (fears, misconceptions, mistrust etc) have been overcome, engagement is a lot easier and then maintained?

To what extent have members of your community changed their views about online services /adapted to using online services?

What do you feel are the benefits for your community from digital engagement?
